# Ascorbylation of a Reactive Cysteine in the Major Apple Allergen Mal d 1

**DOI:** 10.3390/foods11192953

**Published:** 2022-09-21

**Authors:** Linda Ahammer, Jana Unterhauser, Reiner Eidelpes, Christina Meisenbichler, Bettina Nothegger, Claudia E. Covaciu, Valentina Cova, Anna S. Kamenik, Klaus R. Liedl, Kathrin Breuker, Klaus Eisendle, Norbert Reider, Thomas Letschka, Martin Tollinger

**Affiliations:** 1 Institute of Organic Chemistry, Center for Molecular Biosciences Innsbruck (CMBI), University of Innsbruck, 6020 Innsbruck, Austria; 2 Department of Dermatology, Venerology and Allergology, Medical University of Innsbruck, 6020 Innsbruck, Austria; 3 Department of Dermatology, Venerology and Allergology, Central Teaching Hospital, 39100 Bolzano, Italy; 4 Department of Applied Genomics and Molecular Biology, Laimburg Research Centre, 39040 Auer, Italy; 5 Institute of General, Inorganic and Theoretical Chemistry, Center for Molecular Biosciences Innsbruck (CMBI), University of Innsbruck, 6020 Innsbruck, Austria

**Keywords:** chemical modification, conformational epitope, *Malus domestica*, nuclear magnetic resonance, protein structure

## Abstract

The protein Mal d 1 is responsible for most allergic reactions to apples (*Malus domestica*) in the northern hemisphere. Mal d 1 contains a cysteine residue on its surface, with its reactive side chain thiol exposed to the surrounding food matrix. We show that, in vitro, this cysteine residue is prone to spontaneous chemical modification by ascorbic acid (vitamin C). Using NMR spectroscopy and mass spectrometry, we characterize the chemical structure of the cysteine adduct and provide a three-dimensional structural model of the modified apple allergen. The S-ascorbylated cysteine partially masks a major IgE antibody binding site on the surface of Mal d 1, which attenuates IgE binding in sera of apple-allergic patients. Our results illustrate, from a structural perspective, the role that chemical modifications of allergens with components of the natural food matrix can play.

## 1. Introduction

Allergic reactions to apples most frequently affect individuals who already suffer from birch pollen allergy, caused by the protein Bet v 1. This allergic reaction is triggered by immunologic cross-reactivity of Bet v 1-specific IgE antibodies with a structurally homologous protein in apples, Mal d 1. In some countries, more than 70% of all individuals sensitized to birch pollen are affected by cross-reactions to Mal d 1 [1]. Mal d 1 is a cytosolic protein that is present in the flesh, skin and seeds of apples and belongs to a group of 10 pathogenesis-related (PR) proteins whose expression is upregulated in plants in response to biotic and abiotic stress. Dozens of isoforms of Mal d 1 have been identified [2], and the three-dimensional structure of the isoform Mal d 1.0101, initially cloned from ‘Granny Smith’ apples, was determined [3].

Depending on cultivar, storage conditions and harvest years, typically between 1–50 μg of Mal d 1 per gram are present in the flesh of apples, and even higher concentrations have been found in the peel [4]. While Mal d 1 has been identified as the causative agent for the observed allergic reactions, a clear correlation between allergen content and allergic potential of different apple cultivars has not been observed [4,5]. This is, in part, related to the fact that the isoform composition differs significantly between cultivars, and allergenic potential is isoform-specific [5,6]. In addition, interactions of Mal d 1 with the surrounding food matrix on a molecular level can potentially play a significant role.

The canonical PR-10 fold consists of a β-sheet and α-helices that surround an internal cavity, which is capable of promiscuously binding naturally occurring, bioactive compounds with variable affinities [7]. The immunologic consequences of these non-covalent interactions are currently the subject of intense research [8]. In addition, various covalent modifications of food allergens have been reported, including N-glycosylation and Maillard reactions with sugars, which commonly occur during food processing and substantially modulate the observed immunologic properties of these allergens [9].

In apples, ascorbate represents one of the most health-promoting constituents present in considerable quantity. Apples contain 30 mg or more of ascorbate per 100 g of fresh fruit, with substantial variation between different cultivars, exceeding the concentration of Mal d 1 by at least three orders of magnitude (i.e., molar excess) [10]. Moreover, ascorbate is present in almost all compartments of plant cells, including the cytosol [11], where apple allergens are located, and quite commonly, ascorbate is added as a preservative to apple products. The reactivity of a minor apple allergen, Mal d 2, a thaumatin-like protein, towards ascorbate has been studied in detail [12]. Even prolonged incubation of Mal d 2 with ascorbate, however, did not affect the immunological properties of this particular protein.

Interestingly, recent studies established a distinct reactivity of peptides towards ascorbate [13,14]. It was shown that the oxidized form of ascorbate, dehydroascorbate, has a propensity to spontaneously react with the reactive thiol group of cysteines under acidic and neutral conditions, leading to S-ascorbylation of the cysteine side chain. The major apple allergen Mal d 1 contains a single, surface-exposed cysteine residue that is conserved in numerous isoforms. In the present work, the propensity of Mal d 1 to undergo S-ascorbylation is investigated in detail, a structural model of chemically modified Mal d 1 is presented, and the effect of S-ascorbylation on the potential to bind IgE antibodies is characterized in vitro.

## 2. Materials and Methods

Mal d 1 isoforms were recombinantly produced in E. coli and purified by anion exchange chromatography and size-exclusion chromatography as described [15,16]. The molecular weight of Mal d 1 is 17.5 kDa, and its pI is 5.68. S-ascorbylation was achieved as follows: For mass spectrometry, Mal d 1 in 10 mM sodium phosphate buffer pH 6.9 was incubated with a 15-fold molar excess of L-ascorbic acid (Sigma-Aldrich) for seven days at 25 °C, resulting in ≈50% yield. To increase the efficiency of this reaction, we adapted this protocol by switching to oxidized ascorbate. Oxidation of ascorbate was obtained by pre-incubating 10 mg/mL (57 mM) L-ascorbic acid in 10 mM sodium phosphate buffer pH 6.9 at 30 °C for 6–7 days. The oxidation reaction was monitored by recording one-dimensional ^1^H and ^13^C NMR spectra. For NMR spectroscopy, modified Mal d 1 samples were prepared by adding 11.4 mmol of the oxidized ascorbate in 200 μL of buffer to a solution of 5.8 mg (0.33 mmol) unlabeled Mal d 1.0101 in 200 μL of the same buffer (10 mM sodium phosphate, pH 6.9), followed by incubation at 25 °C (3–6 days). Excess ascorbate was removed by centrifugal filtration (3 kDa cutoff). ^13^C_6_-labeled L-ascorbic acid (Sigma-Aldrich) was used to site-specifically isotope label the protein modification, using the same protocol. For ELISA of Mal d 1 isoforms, the incubation times were reduced to one hour at room temperature and overnight at 4 °C to avoid degradation. The progress of the ascorbylation reaction can be monitored using native polyacrylamide gel electrophoresis (Figure S1).

NMR experiments were carried out at 25 °C on 500 MHz and 700 MHz NMR spectrometers equipped with room-temperature triple-resonance probes, using samples containing 0.4–0.6 mM protein in 91% H_2_O/9% D_2_O (*v*/*v*) or in 99.9% D_2_O, at pH 6.9, 10 mM sodium phosphate, respectively. Resonance assignments for Mal d 1.0201 and ascorbylated Mal d 1.0101 were obtained using triple-resonance NMR experiments as previously described [17,18].

Mass spectroscopy was performed using a 7 Tesla Fourier transform ion cyclotron resonance (FT-ICR) mass spectrometer with an electrospray ionization (ESI) source. For MS experiments, 200 μg protein was concentrated to a volume of 50 μL by centrifugal filtration (5 kDa cutoff), and 450 μL of 100 mM ammonium acetate was added. This procedure was repeated five times, followed by five additional cycles using Milli-Q water. MS samples were diluted to yield 1 μM protein in methanol/water 1:1 (*v*/*v*) containing 1% acetic acid.

IgE ELISA experiments were performed as described [19], using sera from patients with a birch pollen-related apple allergy [20,21]. The use of serum samples for this study was approved by the ethics committee of the Medical University of Innsbruck (identification code: 1116/2017). Signed informed consent was obtained from all patients, and samples were analyzed in an anonymized manner. Identical amounts of recombinantly produced isoforms Mal d 1.0101 and Mal d 1.0201 were used in all ELISA experiments.

The structural model of S-ascorbylated Mal d 1 was obtained by molecular dynamics in explicit solvent as described [3]. Parameters for the chemical modification at Cys107 were based on a generalized amber force field (GAFF) [22], and partial charges were fitted using the restrained electrostatic potential (RESP) procedure [23]. The structure of unmodified Mal d 1 (5MMU) was used as a starting structure. Simulations were carried out with positional restraints on all residues except for the Cys107 modification, for which NOE-derived distance restraints were applied using the experimental NOE interproton distances. To efficiently generate a diverse conformational ensemble, we ran 13 parallel MD simulations of 100 ns length each, totaling 1.3 µs of simulation time. Twenty representative structures were extracted using density-based DBSCAN clustering.

## 3. Results

### 3.1. Identification and Characterization of Mal d 1 Ascorbylation

**Figure 1:**
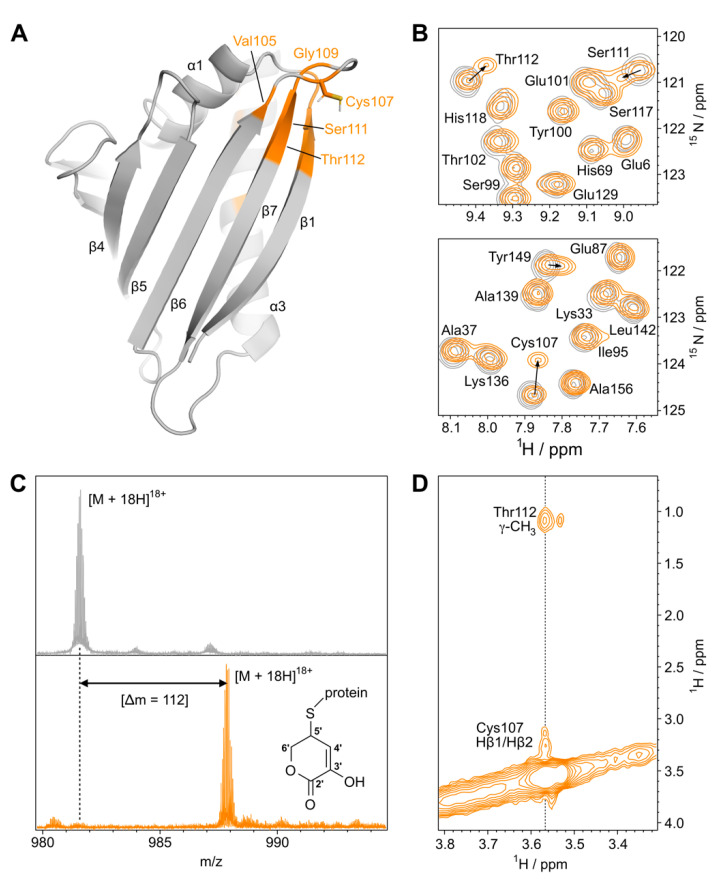
Ascorbylation of Cys107 in Mal d 1 (isoform Mal d 1.0101): (**A**) Backbone representation of Mal d 1 (pdb: 5MMU). Amino acid residues with backbone amide ^1^H^15^N chemical shift perturbations upon reaction with L-ascorbate exceeding 0.2 ppm are colored in orange. The side chain of the surface-exposed residue Cys107 is shown. (**B**) Sections from backbone amide ^1^H^15^N-HSQC spectra of Mal d 1 before (gray) and after (orange) incubation with L-ascorbate (15-fold excess) for seven days at 25 °C. From the observed intensities in the ^1^H^15^N-HSQC spectrum, we estimate that ascorbylation is ≈50% complete after one week. (**C**) ESI mass spectrum of 18-fold positively charged Mal d 1, recorded on an FT-ICR mass spectrometer before (top) and after incubation with L-ascorbate at 25 °C for two weeks (bottom). The mass shift, Δm, indicates the formation of a covalent adduct with C_5_H_4_O_3_. (**D**) Section from a two-dimensional ^13^C-filtered NOESY spectrum of Mal d 1 after reaction with ^13^C-labeled L-ascorbate (700 MHz, 120 ms mixing time). Assignments are shown for NOE cross peaks to the ^13^C-bound proton H5′ at 3.57 ppm.

In the three-dimensional solution structure of Mal d 1, the single cysteine residue at position 107 is exposed on the protein surface (Figure 1A). Cys107 is located in a loop that connects two strands of the curved β-sheet and has its reactive side chain protruding into the surrounding solvent. In a first step, we probed the reactivity of Mal d 1 toward L-ascorbate using solution NMR spectroscopy. The addition of L-ascorbate to samples of Mal d 1 did not have an immediate impact on the backbone amide ^1^H^15^N-HSQC spectrum of this protein (Figure S2), even at 15-fold excess, indicating that, unlike other amphiphilic compounds of similar size [24], L-ascorbate does not bind to the internal cavity of this protein. However, incubation with L-ascorbate for several days initiated a slow but defined change of the backbone amide ^1^H^15^N-HSQC spectrum (Figure 1B). Changes in ^1^H^15^N resonance positions were only observed for a limited number of amino acids in Mal d 1, while most backbone amide resonances remained unaffected (Figure S2). Chemical shift changes exceeding 0.2 ppm were found for amino acids in the five-residue loop that harbors Cys107 and the surrounding β-strands (β6 and β7), covering the entire stretch between Val105 in strand β6 and Ile113 in strand β7. Chemical shift changes were also observed at the tip of strand β1, which is adjacent to strand β7, involving residues Phe9, Thr10, Ser11 and Glu12, and for three amino acids (His140, Ile146 and Tyr149) in the C-terminal helix α3. Helix α3 packed to hydrophobic side chains in strands β1 and β7 and had contact with residues Phe9 and Ile113, respectively. Notably, all backbone amides with resonance shifts exceeding 0.2 ppm were located less than 10 Å away from the solvent-exposed side chain of Cys107. Since the vast majority of Mal d 1 resonances in ^1^H^15^N-HSQC spectra were, however, not affected by ascorbate, these data clearly indicate that the three-dimensional structure and the hydrogen-bonded backbone of the protein were not perturbed.

We next probed the reactivity of Mal d 1 toward L-ascorbate using ESI FT-ICR mass spectrometry (Figure 1C), which indicated the formation of an adduct between Mal d 1 and a five-carbon fragment of ascorbate, corresponding to a mass shift of +112 Da (C_5_H_4_O_3_). This mass shift has previously been observed for a model protein, where it was attributed to the attachment of a six-membered ring structure to the side chain sulfur atom of cysteine that contains five carbon atoms from ascorbate [14]. To characterize the adduct between Mal d 1 and ascorbate in detail, we employed heteronuclear NMR spectroscopy. Using ^13^C-labeled L-ascorbate as the substrate, site-specific isotope labeling of the adduct in the apple allergen was achieved, and a two-dimensional ^1^H^13^C-HSQC spectrum was recorded (Figure S3). The observed chemical shifts of non-exchangeable protons and their adjacent carbon nuclei were in accord with the structure shown in Figure 1C, with 3-hydroxy-5,6-dihydro-2H-pyran-2-one attached to a cysteine residue. ^1^H and ^13^C resonances at 3.5–3.6 ppm and 29.0 ppm, respectively, were within the chemical shift range that is expected for H5′/C5′, with the distinctive downfield shifts caused by the formation of a thioether as a result of S-ascorbylation. ^1^H and ^13^C resonances in the low-field region of the ^1^H^13^C-HSQC spectrum (6.5 ppm ^1^H and 110 ppm ^13^C) suggest that, under our experimental conditions, the enolic form of the six-membered ring was present, which is shown in Figure 1C.

### 3.2. Structure of Ascorbylated Mal d 1

The orientation of the ascorbate-derived ring structure with respect to the Mal d 1 scaffold was again evaluated by NMR. The site-specifically ^13^C-labeled adduct facilitated the use of ^13^C-filtered nuclear Overhauser effect (NOE) experiments to probe atomic distances between the chemical modification and the protein (Figure 1D). For ^1^H at position 5′ of the ^13^C-labeled ring structure (^1^H chemical shift: 3.57 ppm), NOE cross peaks were visible in the spectrum. Apart from short-range NOEs between H5′ and the side chain Hβ nuclei of Cys107, the data indicated spatial proximity of H5′ to the side chain methyl group (γ-CH_3_) of amino acid Thr112 (^1^H chemical shift: 1.09 ppm) in strand β7.

**Figure 2:**
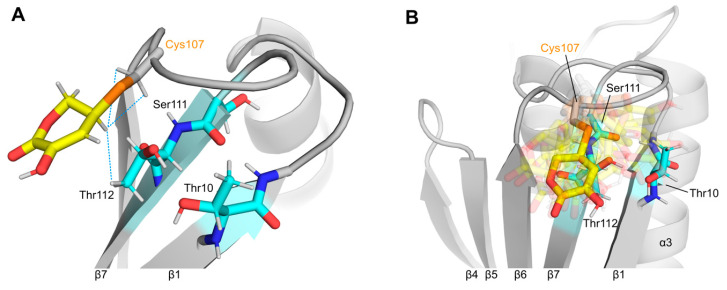
Structural model of S-ascorbylated Mal d 1.0101: (**A**) Representative structure showing the orientation of the covalently bound six-membered ring in yellow. NOE contacts to surface-exposed amino acid residues in Mal d 1 are indicated as blue dotted lines. Residues forming the conformational IgE epitope Thr10-Ser111-Thr112 are highlighted (cyan). (**B**) Orientational variability of the ascorbate-derived ring structure in the 20 MD structures. The representative orientation shown in (**A**) is non-transparent.

We used the experimental NOE data as input restraints for molecular dynamics (MD) simulations to visualize the orientation of the ascorbate-derived ring structure on the surface of Mal d 1 (Figure 2). In the MD structural ensemble, the six-membered ring packed to an amphiphilic patch on the protein surface formed by the curved β-sheet and connecting loops. H5′ was 2.5–2.8 Å away from the side chain Hβ atoms of Cys107 and 2.5–4.5 Å (inter-proton distance) from the side chain γ-CH_3_ group of Thr112 in all conformers. Figure 2B illustrates that the orientation of the ascorbate-derived ring structure on the protein surface was fairly flexible. While it was centered directly above amino acids Ser111 and Thr112 in strand β7 in most conformers, it extended to the tips of strands β1 (Phe9, Thr10) and β6 (Val105) in other conformers of the ensemble. This is in good agreement with the observed chemical shift perturbations in this part of the Mal d 1 structure (Figure 1). Notably, the experimental NOE data were relatively sparse, which may have been caused by conformational heterogeneity, as present also in the structural ensemble [25].

### 3.3. Effect of Mal d 1 Ascorbylation on Antibody Binding

**Figure 3:**
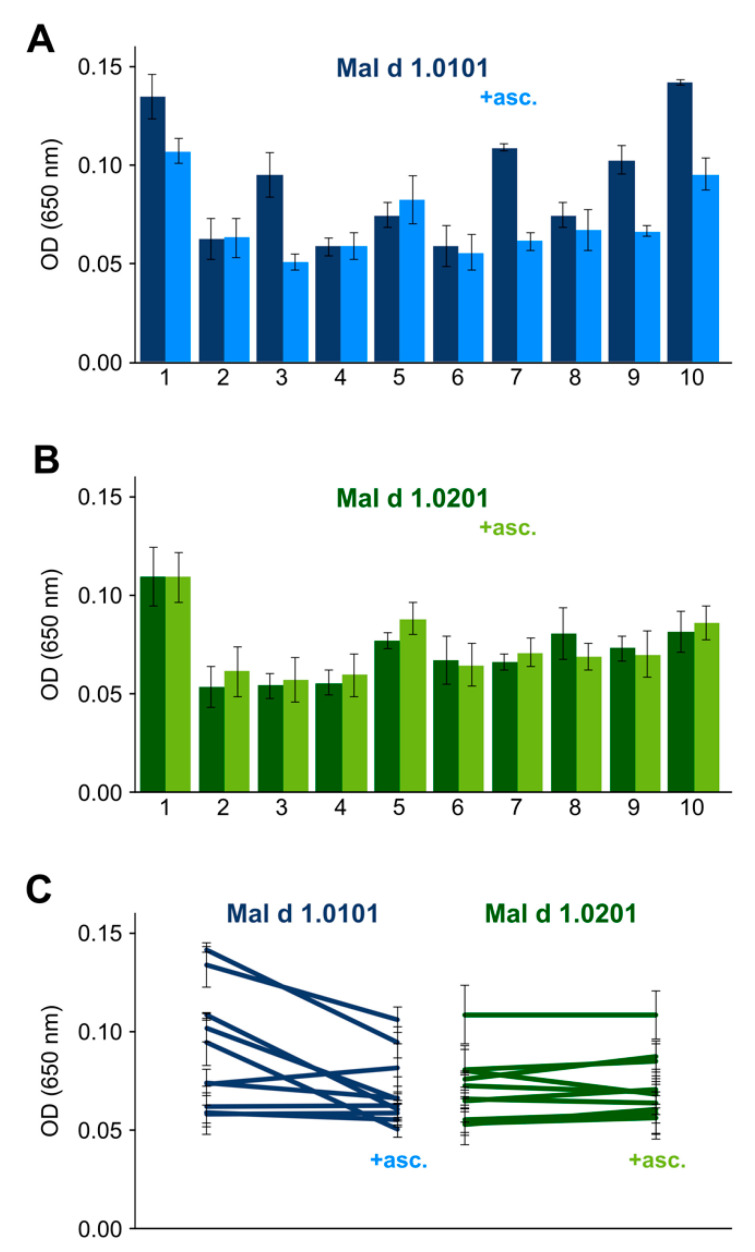
Effect of cysteine S-ascorbylation on IgE binding to Mal d 1: (**A**) IgE reactivity to the plate-bound isoform Mal d 1.0101, as measured by ELISA using blood sera of 10 birch pollen- and apple-allergic patients. Data are shown for recombinantly produced allergen before (dark blue) and after incubation with oxidized L-ascorbate (light blue). Identical amounts of allergen were used in all experiments. Depending on the available sera, either one or two experiments in triplicates were performed (see Tables S1 and S2). Average OD values are shown. Error bars represent standard deviations. (**B**) ELISA data for the isoform Mal d 1.0201, which lacks surface-exposed cysteine residues, before (dark green) and after incubation with L-ascorbate (light green). (**C**) Individual, patient-specific trends illustrate changes in IgE reactivities upon S-ascorbylation in the isoform Mal d 1.0101.

We evaluated the effect of S-ascorbylation on antibody binding by IgE ELISA using blood sera from 10 patients who had previously been identified as being allergic to both birch pollen and apples [20]. Figure 3 shows the IgE reactivities to Mal d 1 with and without the chemical modification being present (the experimental data are compiled in Tables S1 and S2). It is evident from these experiments that S-ascorbylation of Mal d 1.0101 indeed had a measurable effect on the IgE binding potential. This effect appears to be patient-specific, with IgE binding being significantly attenuated by ascorbylation for some patients (ca. 50% of the cohort), while it remained unaffected for the remainder of the cohort.

We also assessed the IgE reactivity of the isoform Mal d 1.0201. This particular isoform shares 90.5% sequence identity with Mal d 1.0101, and NMR spectroscopic characterization shows that it was fully folded under the experimental conditions used (Figure S4). In Mal d 1.0201, which does not contain cysteines, the surface-exposed amino acid position 107 is occupied by a serine, and S-ascorbylation was not possible. The experimental ELISA data (Figure 3) show that this isoform, too, was recognized and bound by IgE in the blood sera of all patients in the cohort. In contrast to Mal d 1.0101, however, IgE reactivities toward Mal d 1.0201 were not attenuated after incubation with L-ascorbate.

## 4. Discussion and Conclusions

In vitro incubation of Mal d 1 with ascorbate induced S-ascorbylation of the surface-exposed cysteine residue at position 107. As a result, a six-membered ring structure, which derived from oxidized ascorbate, was covalently attached to the side chain of this amino acid. Intriguingly, structural analysis revealed that this ring structure was located near residues Thr10, Ser111 and Thr112. These amino acids have previously been identified as being part of a known IgE epitope in Mal d 1 [3]. Mutation of these residues in Mal d 1 significantly reduced IgE binding in vitro, and skin prick tests in apple-allergic patients revealed a measurably lower ability to induce skin reactions in vivo [26,27]. In our structural model, the covalently bound ring structure packed to the amphiphilic side chain of Thr112 and the backbone amide of Ser111 and partly covered and masked the side chain of Thr10.

Epitope masking can potentially hinder access of antibodies and cause a reduction in conformational epitopes that are available for binding. Our data show that, upon S-ascorbylation, the IgE binding reactivity of Mal d 1 was reduced, but it did not disappear. Mal d 1, like other PR-10 allergens, contains more than a single conformational epitope on its surface, and several amino acids outside the Thr10-Ser111-Thr112 epitope have been described as being critical for IgE binding to Mal d 1 (e.g., Ile30, Thr57) [26,28]. In the three-dimensional structure of Mal d 1, these amino acids are more than 25Å (arc length) away from the reactive site at Cys107 and, therefore, not within the surface area that is covered by the ascorbate-derived ring structure.

S-ascorbylation requires the oxidized form of ascorbate, dehydroascorbate, and degradation products thereof to be present [29]. In vitro, the rate at which S-ascorbylation occurs is significantly enhanced when ascorbate is previously oxidized. In fresh apples, roughly 30% of total ascorbate is present in the form of dehydroascorbate [30]. This balance shifts towards the oxidized form during long-term storage, presumably due to oxidative stress in the cell. Likewise, ascorbate is oxidized upon exposure of the fruit to air by cutting and during food processing. Whether Mal d 1 is (partly) S-ascorbylated in apples or becomes S-ascorbylated during food processing is currently unknown. Proteomic profiles have only recently been reported for several apple genotypes, providing valuable insight into the isoform composition [2]. Proteomic analysis does, however, require reducing agents to avoid cysteine-mediated dimerization, which presumably also inhibits or even reverts S-ascorbylation.

The potential impact of food processing on the allergenicity of food has been extensively studied [31]. Interestingly, the surface-exposed cysteine residue at position 107 is not present in all Mal d 1 isoforms. While Cys107 is conserved within the Mal d 1.01 cluster (Mal d 1.0101–Mal d 1.0109), it is substituted by serine in the Mal d 1.02, Mal d 1.03 and Mal d 1.04 clusters. Differential reactivity of Mal d 1 isoforms toward components of the food matrix, as observed in our study, is thus to be expected and may well contribute to the discrepancy between Mal d 1 content and apple tolerability. The data presented here provide a structure-based model for how interactions of allergenic proteins with food matrix components may play a role in food allergy.

## Data Availability

The data presented in this study are available on request from the corresponding author.

## Supplementary Materials

The following supporting information can be downloaded at: www.mdpi.com/article/10.3390/foods11192953/s1, Figure S1: Native polyacrylamide gel electrophoresis data for Mal d 1.0101 showing the progress of ascorbylation; Figure S2: Full backbone amide ^1^H^15^N-HSQC spectra of Mal d 1.0101 in the presence of ascorbate; Figure S3: ^1^H^13^C-HSQC spectra of ascorbylated Mal d 1.0101; Figure S4: Backbone amide ^1^H^15^N-HSQC spectra of isoforms Mal d 1.0101 and Mal d 1.0201; Table S1: ELISA data for Mal d 1.0101; Table S2: ELISA data for Mal d 1.0201.
