# Supplementary material for: Ascorbylation of a Reactive Cysteine in the Major Apple Allergen Mal d 1
Source: Foods. 2022 Sep 21;11(19):2953. doi: 10.3390/foods11192953 (PMC9562000; doi:10.3390/foods11192953)
Supplement: Supplementary file 1 [file foods-11-02953-s001.zip › foods-1878415-supplementary.pdf]

Supporting Information

Ascorbylation of a Reactive Cysteine in the Major Apple Allergen Mal d 1

Linda Ahammer, Jana Unterhauser, Reiner Eidelpes, Christina Meisenbichler, Bettina Nothegger, Claudia E. Covaciu, Valentina Cova, Anna S. Kamenik, Klaus R. Liedl, Kathrin Breuker, Klaus Eisendle, Norbert Reider, Thomas Letschka and Martin Tollinger

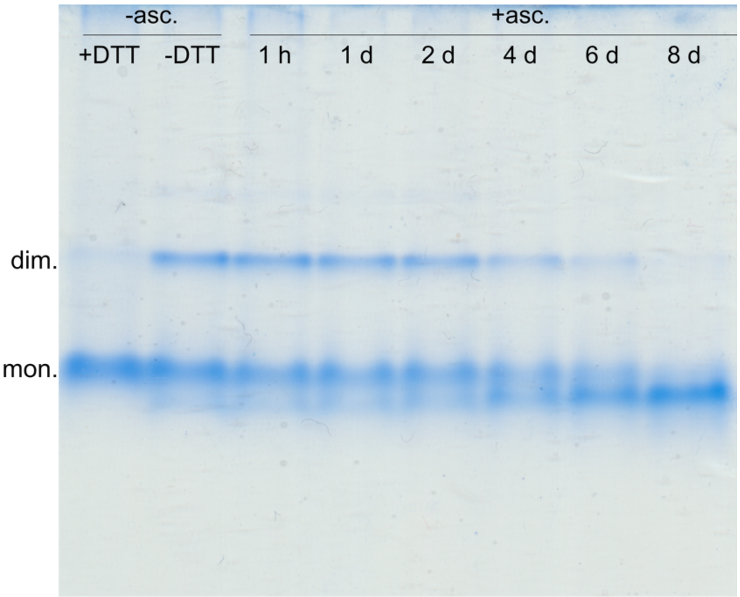

Figure S1: Native polyacrylamide gel electrophoresis (PAGE) for monitoring the effect of incubating Mal d 1 with L-ascorbate. Experimental conditions: 0.025mM Mal d 1.0101 was incubated with a 10-fold molar excess of L-ascorbate in 10 mM sodium phosphate buffer pH 6.9 at 25°C. A 15% polyacrylamide gel was used for PAGE. Samples taken after 1 hour, 2, 4, 6 and 8 days (lanes 3-8) show that during this time the Mal d 1 band gradually shifts. Lanes 1 and 2 show that dimerization of Mal d 1, which was previously reported (Kaeswurm, J.A.H.; Nestl, B.; Richter, S.M.; Emperle, M.; Buchweitz, M.: Purification and characterization of recombinant expressed apple allergen Mal d 1. *Methods Protoc.* **2021**, *4*, 3), can be reverted by the addition of 1mM 1,4-dihiothreitol (DTT).

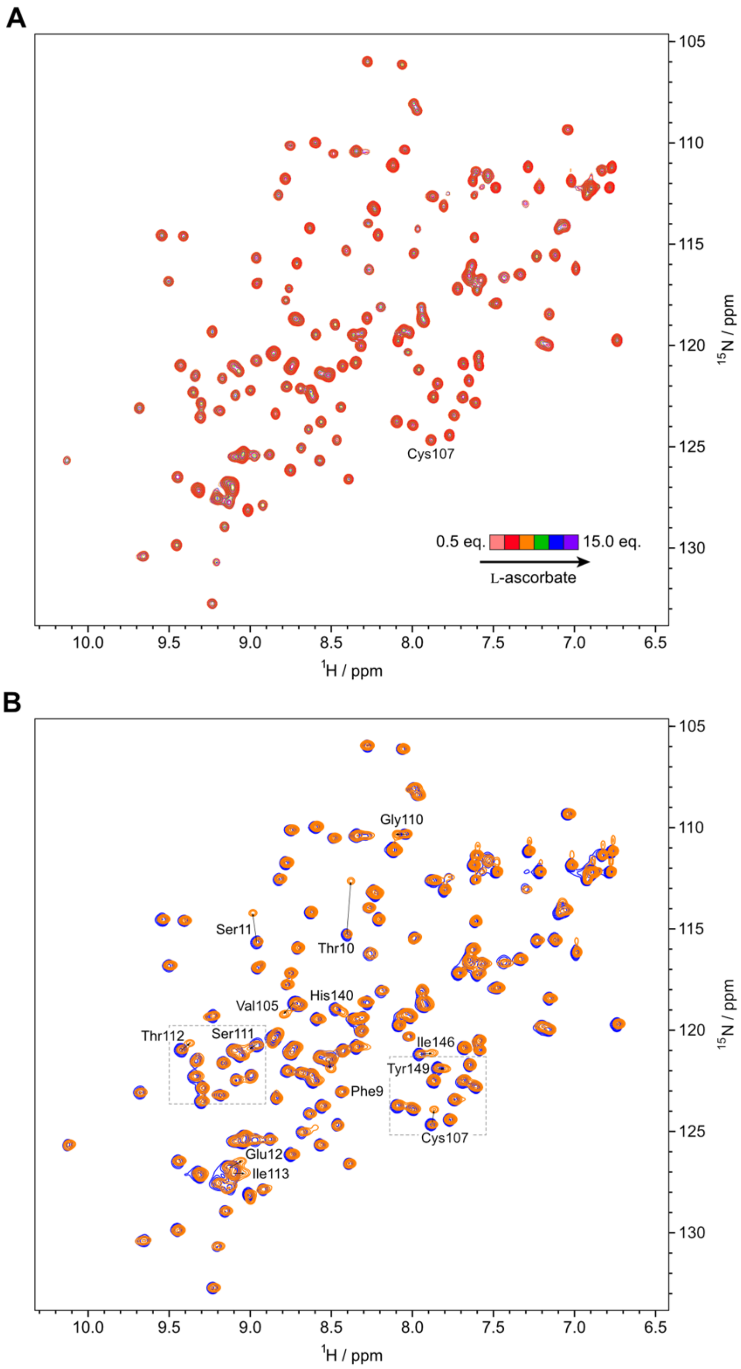

Figure S2: (**A**) Backbone amide ^{1}H^{15}N-HSQC spectra of ^{15}N-labeled Mal d 1.0101 in the presence of variable amounts of L-ascorbate (up to 15-fold molar excess). All spectra were recorded immediately after addition of L-ascorbate, 500 MHz. Sample conditions: 0.4 mM Mal d 1, 10 mM sodium phosphate buffer pH 6.9, 9% D_2O, 25 °C. Concentrations of L-ascorbate: 0.2 mM, 0.4 mM, 0.8 mM, 2.0 mM, 4.0 mM, 6.0 mM. To ensure identical experimental conditions in all titration steps, L-ascorbate was dissolved in the same buffer as the protein and the pH was adjusted to pH 6.9 before addition. (**B**) ^{1}H^{15}N-HSQC spectra before (blue) and after (orange) incubation with L-ascorbate (15-fold molar excess) for one week at 25 °C. Residues with ^{1}H/^{15}N chemical shift changes exceeding 0.2 ppm are labeled. Gray dashed boxes are drawn for sections of the spectrum that are shown in Figure 1.

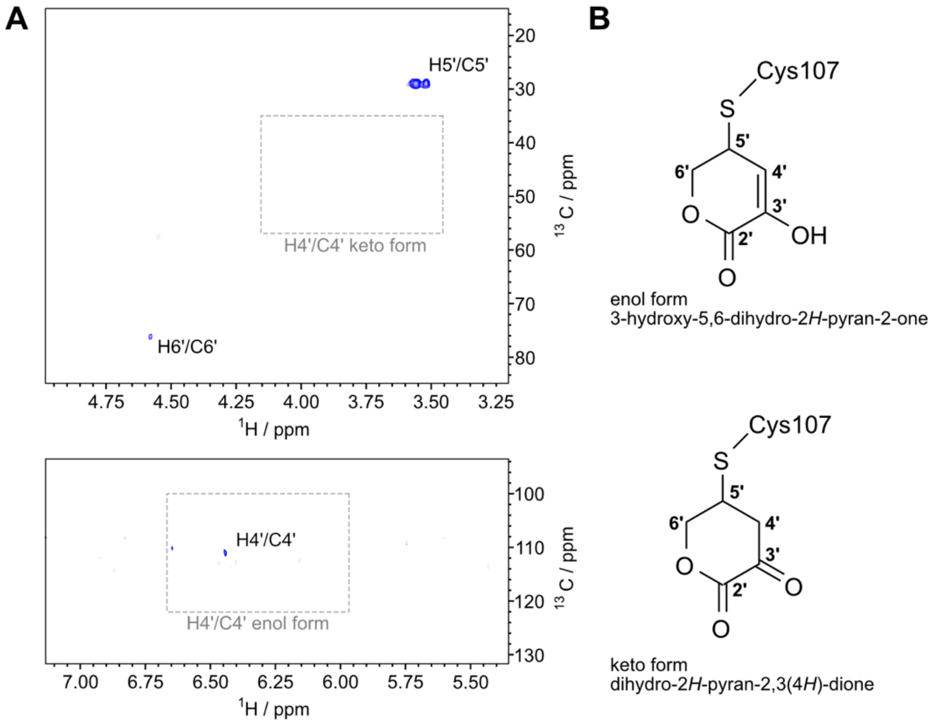

Figure S3: (**A**) Sections from a two-dimensional ^{13}C-filtered ^{1}H^{13}C-HSQC spectrum of Mal d 1 after reaction with $^{13}C_6$-labeled L-ascorbate, recorded at 700 MHz, in 99.9% D_2O, at pH 6.9, 10 mM sodium phosphate. The observed resonances in the aliphatic (top) and olefinic (bottom) region of the ^{1}H-^{13}C-HSQC spectrum are consistent with the enolic form (**B**, top) but not with the keto form (**B**, bottom) of the six-membered ring structure, which was proposed for glutaredoxin (Flandrin, A.; Allouche, S.; Rolland, Y.; McDuff, F. O.; Richard Wagner, J. R., Klarskov, K.: Characterization of dehydroascorbate-mediated modification of glutaredoxin by mass spectrometry. *J. Mass Spectrom.* **2015,** *50* (12), 1358-1366). The observation of two distinct ^{1}H resonances for H5' is indicative for the presence of two diastereomers. Resonance assignments for H4'/C4', H5'/C5' and H6'/C6' are based on the observed ^{1}H and ^{13}C chemical shifts. Resonance intensities for H4'/C4' and H6'/C6' are low, possibly due to conformational heterogeneity.

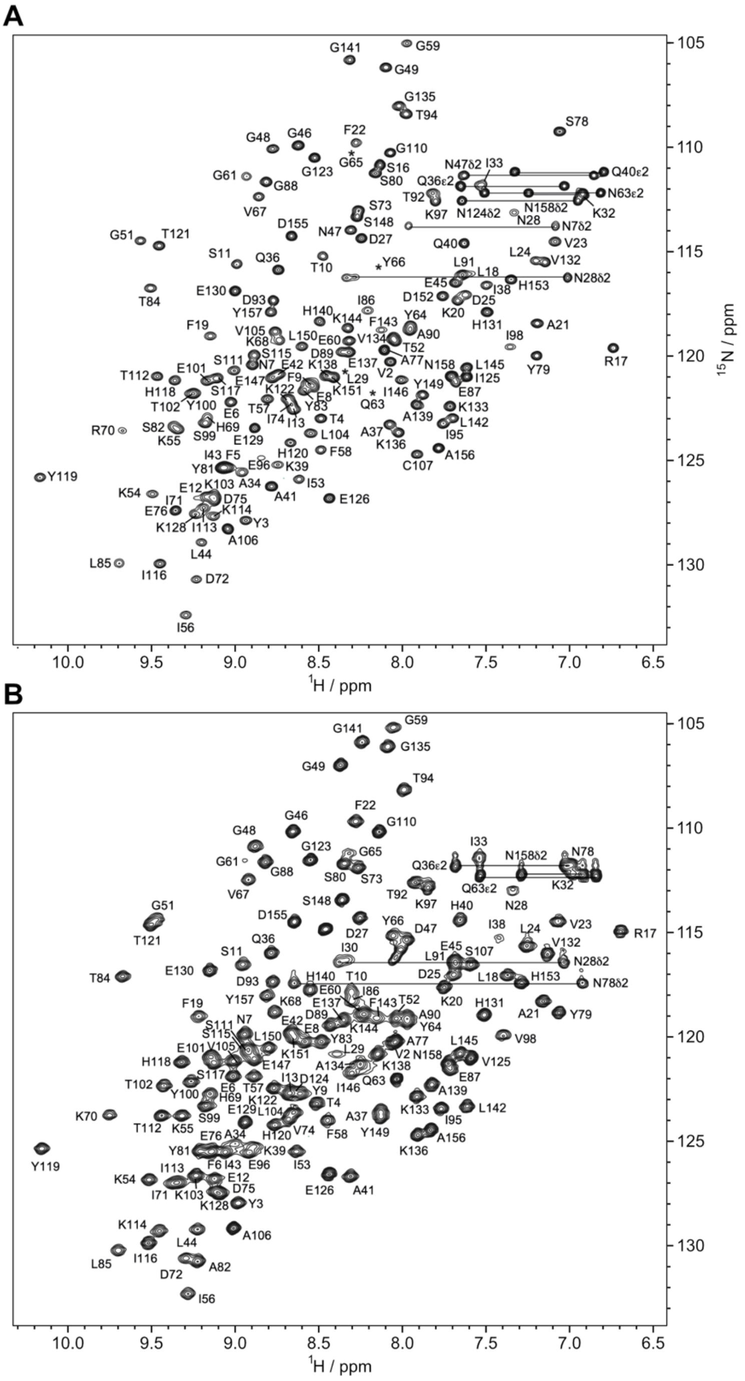

C

```
Mal d 1.0101      GVYTFENEFT SEIPPSRLFK AFVLDADNLI PKIAPQAIKQ AEILEGNGGP GTIKKITFGE  60
Mal d 1.0201      GVYTFENEYT SEIPPPRLFK AFVLDADNLI PKIAPQAIKH AEILEGDGGP GTIKKITFGE  60
                  ********:* ***** **** ********** *********: ******:*** **********

Mal d 1.0101      GSQYGYVKHR IDSIDEASYS YSYTLIEGDA LTDTIEKISY ETKLVACGSG STIKSISHYH 120
Mal d 1.0201      GSQYGYVKHK IDSVDEANYS YAYTLIEGDA LTDTIEKVSY ETKLVASGSG SIIKSISHYH 120
                  *********: ***:***.** *:******** *******:** ******.*** * ********

Mal d 1.0101      TKGNIEIKEE HVKVGKEKAH GLFKLIESYL KDHPDAYN  158
Mal d 1.0201      TKGDVEIKEE HVKAGKEKAH GLFKLIESYL KGHPDAYN  158
                  ***::***** ***.****** ********** *.******
```

Figure S4: Backbone amide $^{1}H^{15}N$-HSQC spectra and resonance assignments of isoforms (**A**) Mal d 1.0101 and (**B**) Mal d 1.0201 of the major apple allergen. Sample conditions: 0.5 mM ^{15}N-labeled protein, 10 mM sodium phosphate buffer pH 6.9, 7.0 mM L-ascorbate, 9 % D_2O. (**C**) Comparison of amino acid sequences of Mal d 1.0101 and Mal d 1.0201. Stars indicate identical residues, while colons and dots indicate conservation of residues with strongly and weakly similar properties, respectively.

	Mal d 1.0101							
	1	2	3	4	5	6	**mean**	**std. dev.**
**patient 1**	0.131	0.120	0.127	0.145	0.132	0.150	**0.134**	**0.011**
**patient 2**	0.063	0.062	0.081	0.051	0.054	0.062	**0.062**	**0.010**
**patient 3**	0.082	0.105	0.097				**0.095**	**0.012**
**patient 4**	0.054	0.058	0.063				**0.058**	**0.005**
**patient 5**	0.071	0.070	0.082				**0.074**	**0.007**
**patient 6**	0.052	0.053	0.071				**0.059**	**0.011**
**patient 7**	0.107	0.110	0.109				**0.109**	**0.002**
**patient 8**	0.076	0.067	0.080				**0.074**	**0.007**
**patient 9**	0.101	0.110	0.096				**0.102**	**0.007**
**patient 10**	0.143	0.141	0.197				**0.142**	**0.001**

	Mal d 1.0101 + asc.							
	1	2	3	4	5	6	**mean**	**std. dev.**
**patient 1**	0.115	0.103	0.113	0.102	0.099	0.107	**0.107**	**0.006**
**patient 2**	0.073	0.057	0.067	0.049	0.057	0.073	**0.063**	**0.010**
**patient 3**	0.055	0.050	0.047				**0.051**	**0.004**
**patient 4**	0.059	0.052	0.065				**0.059**	**0.007**
**patient 5**	0.077	0.073	0.096				**0.082**	**0.012**
**patient 6**	0.048	0.053	0.065				**0.055**	**0.009**
**patient 7**	0.059	0.066	0.058				**0.061**	**0.004**
**patient 8**	0.061	0.060	0.079				**0.067**	**0.011**
**patient 9**	0.065	0.064	0.069				**0.066**	**0.003**
**patient 10**	0.103	0.095	0.087				**0.095**	**0.008**

Table S1: IgE reactivity to the isoform Mal d 1.0101, measured by ELISA using blood sera of 10 birch pollen and apple allergic patients, with and without prior incubation of the allergen with oxidized ascorbate. OD values recorded at a wavelength of 650 nm are reported. For patients 1 and 2, two experiments in triplicates (columns 1-3 and 4-6) were performed, while for patients 3-10 one experiment in triplicates (columns 1-3) was performed. For patient 10, one OD value (shown in gray) was identified as outlier (>95% confidence) and excluded from further analysis. Mean values and standard deviations were used for generating Figure 3. Mean OD values for negative serum samples were 0.045 (standard deviation: 0.003) before and 0.047 (0.004) after S-ascorbylation.

	Mal d 1.0201							
	1	2	3	4	5	6	**mean**	**std. dev.**
**patient 1**	0.105	0.096	0.095	0.110	0.112	0.136	**0.109**	**0.015**
**patient 2**	0.058	0.056	0.069	0.042	0.044	0.049	**0.053**	**0.010**
**patient 3**	0.047	0.055	0.059				**0.054**	**0.006**
**patient 4**	0.049	0.056	0.061				**0.055**	**0.006**
**patient 5**	0.081	0.074	0.074				**0.076**	**0.004**
**patient 6**	0.053	0.077	0.069				**0.066**	**0.012**
**patient 7**	0.070	0.063	0.063				**0.065**	**0.004**
**patient 8**	0.081	0.067	0.093				**0.080**	**0.013**
**patient 9**	0.073	0.079	0.066				**0.073**	**0.007**
**patient 10**	0.093	0.076	0.074				**0.081**	**0.010**

	Mal d 1.0201 + asc.							
	1	2	3	4	5	6	**mean**	**std. dev.**
**patient 1**	0.112	0.103	0.127	0.099	0.094	0.117	**0.109**	**0.012**
**patient 2**	0.074	0.054	0.080	0.056	0.048	0.053	**0.061**	**0.013**
**patient 3**	0.046	0.056	0.068				**0.057**	**0.011**
**patient 4**	0.055	0.051	0.071				**0.059**	**0.011**
**patient 5**	0.089	0.095	0.079				**0.088**	**0.008**
**patient 6**	0.053	0.066	0.074				**0.064**	**0.011**
**patient 7**	0.068	0.079	0.065				**0.071**	**0.007**
**patient 8**	0.070	0.061	0.074				**0.068**	**0.007**
**patient 9**	0.063	0.083	0.063				**0.070**	**0.012**
**patient 10**	0.095	0.083	0.078				**0.085**	**0.009**

Table S2: IgE reactivity to the isoform Mal d 1.0201, as measured by ELISA using blood sera of 10 birch pollen and apple allergic patients. OD values recorded at a wavelength of 650 nm are reported. For patients 1 and 2 two experiments in triplicates (columns 1-3 and 4-6) were performed, while for patients 3-10 one experiment in triplicates (columns 1-3) was performed. Mean values and standard deviations were used for generating Figure 3.
